# Dr. Drake and the Rush Medical College

**Published:** 1844-12

**Authors:** 


*Dr. Drake and the Rush Medical College.*—It is known to many of our readers that Dr. Drake, of the "Louisville Medical Institute," visited Chicago and the region of the lakes during the past summer, for the very praiseworthy purpose of collecting, by enquiry, materials for his work on the diseases of the Mississippi valley. During his tour he has, from time to time, been in the habit of gratifying the readers of the "Western Journal of Medicine and Surgery," by "Traveling Letters" on medical and miscellaneous subjects. We give below, so much of his letter from this place as relates to our school, but cannot do so without a remark upon one or two points upon which it touches. In reference to the prices of tickets, they are higher in the Rush Medical College than in most of the medical schools of the eastern States.

The lecture fees in the Rush Medical College are $60; Metriculution fee $5, Dissecting ticket $5, Graduation fee $20.

The fee for the entire course of lectures in the "Castleton Medical College" is $50; Metriculation fee $5, Graduation $16. In the "New Hampshire Medical Institution" $50; Matriculation fee $5. In the Berkshire Medical College the same; at Gevena $65; in Willoughby Medical School $58.

It will be seen that our fees are higher than those of most of the medical schools with which with which we come in competition. In the Cleveland Medical School the price of tickets was fixed at $72, but for some reason has been reduced to $50. We confesss that we cannot see any good reason for such a course. We consider it undignified and calculated to injure the profession and the public, and regret that gentlemen of so much character, and physicians of so much reputation as those connected with that school, should have been for a moment induced to take such a course.

If our fees are lower than those of the "Louisville Medical Institute," it is not owing to any disposition on our part to cheapen medical degrees, but to the practices of this region being different from those of Kentucky; and in proof of the seriousness of our intentions in this respect, we point with some pride to the fact that our requirements for graduation are higher than those of the Louisville school, there being no time specified in their annual anouncement during which the student must have pursued his studies, while here three years are required. As their class is large and increasing, we can see no good reason why some effort should not be made to increase the term of study and facilities for instruction:

we think this more important than advancing the price of tickets. We may add that our anticipations in regard to the progress of the school have been happily disappointed, the present class being more than double that of last year, in numbers, and that all appearances are in favor of the rapid advance of the school.

"Chicago is the head of navigation on Lake Michigan; the port of embarkation for travelers from the south-west, who would reach New York or Boston by Mackinac and Detroit; the place of debarkation for travelers going in the opposite direction, and for emigrants from the northern States to Illinois, Iowa, and Missouri. You will not be surprised, then, to learn, that in 13 years, it has risen from a few families, to a population of nearly 10,000; that it revels in high anticipations; and cherishes quite as much ambition as any other precocious American city.

"Now, lest some of our readers, desirous of changing their location, should resolve to put off for Chicago, I feel it my duty to tell them, that she is already abundantly furnished with physicians. Of the exact number I cannot speak, but the *signs* of an adequate supply are conspicuous; and arrangements have been made for keeping it up, by home manufacture. Of this I gave you intimation in my last, expressing some disapprobation of the proposal to manufacture them here, and in some other places, at so small a cost. In the four days which have elapsed since the date of that letter, my opinions have undergone no change, and of course I still think, that as a cutler, who might advertise to make surgical instruments at half price, would not be patronized by our operators, so, surgeons made at half-price, should not, *a priori*, be regarded by the people, as strong and acute. Among the instruments made by the manufacturer of 'cheap goods,' there might now and then be one of excellent temper, and among the alumni of our cheap schools there may be found some of excellent professional temperament, but neither the shops nor the schools should be judged by these exceptions. Justice however, to Dr. Brainard, the enlightened founder of the 'Rush Medical College' of this place, requires me to state, that he himself, in the abstract, does not approve of cheapening medical instruction; but says he was driven into it by the example of the schools in this latitude, from Geneva, in New York, to Fox River, in Illinois, embracing the intermediate establishments of Willoughby, Cleveland, Laport, and Jacksonville. He aud his colleagues, indeed, have it in view, in due time, to advance the price of their tickets. He does not anticipate a very rapid growth of his establishment, and would not, in the infancy of the country, have moved in its organization, but that several towns, which he justly regarded as too small and sequestered, had been made the sites of such institutions. I must confess that this view of the matter is plausible; and that if the patronage which would be distributed among the towns of Laporte, Jacksonville, and St. Charles, can be concentrated on

Chicago, it will be for the benefit of the profession and society at large. Indeed, it must, I think, be admitted, that the towns just named, together with Willoughby, in the State of Ohio, are not places where flourishing medical colleges can be built. West of Pennsylvania and New York, leaving out of view the towns on or near the Ohio, the three points favoring and requiring such seminaries, are Saint Louis, Chicago, and Cleveland. The first is connected with the Lower and Upper Mississippi and with Western Illinois; the second has connection with Northern Indiana, middle and Northern Illinois, Western and Northern Michigan, Wisconsin and Iowa; the third may look to Michigan, North-eastern Indiana, Northern Ohio, North-western Pennsylvania, and Upper Canada; within which various States and Territories, there will, in another age, be a dense population, supplying students enough for three schools, but not for the *eight* which have already announced themselves. Each of the towns which I have mentioned will have population enough to give business to a faculty of teachers, and subjects for the practical anatomist; each will have hospital, where clinical medicine and morbid anatomy may be taught; and each will erect such literary and scientific associations, as will favor research and ambitious study, in both professors and pupils.

"The first annual catalogue of the school of this place, just published, embraces 22 students, which is rather more than the first class in Transylvania, or the Medical College of Ohio, and beyond what might have been expected on a spot, which 15 years ago was but a military post and the site of an Indian Agency. I have made a visit to the edifice of the school—(to be finished in time for the ensuing course of lectures)—which will be commodious and respectable. The money with which the Trustees are building it, has been cheerfully contributed by the citizens of Chicago. The faculty have also commenced a *monthly*, under the title of the "Illinois Medical and Surgical Journal," of which Prof. Blaney is the Editor. Each number contains 16 large and well-printed pages the contents of which I have not had time to study, but observe a large proportion of original matter. Thus Chicago is fairly in the field, and her nearest rival will be St. Louis. I hope they will set an example to their seniors, of honorable bearing in the *certamen gloriæ.*"

